# Comparative analysis of the root transcriptomes of cultivated and wild rice varieties in response to *Magnaporthe oryzae* infection revealed both common and species-specific pathogen responses

**DOI:** 10.1186/s12284-018-0211-8

**Published:** 2018-04-20

**Authors:** Lei Tian, Shaohua Shi, Fahad Nasir, Chunling Chang, Weiqiang Li, Lam-Son Phan Tran, Chunjie Tian

**Affiliations:** 1 Key Laboratory of Mollisols Agroecology, Northeast Institute of Geography and Agroecology, Chinese Academy of Sciences, Changchun, 130102 China; 2 University of Chinese Academy of Sciences, Beijing, 100049 China; 3 School of Life Sciences, Northeast Normal University, Changchun City, Jilin China; 4 Signaling Pathway Research Unit, RIKEN Center for Sustainable Resource Science, 1-7-22, Suehiro-cho, Tsurumi, Yokohama, 230-0045 Japan; 5 Institute of Research and Development, Duy Tan University, 03 Quang Trung, Da Nang, Vietnam; Signaling Pathway Research Unit, RIKEN Center for Sustainable Resource Science, 1-7-22, Suehiro-cho, Tsurumi, Yokohama, 230-0045 Japan

**Keywords:** Cultivated rice, *Magnaporthe oryzae*, Transcriptome analysis, RNA-sequencing, Wild rice

## Abstract

**Background:**

*Magnaporthe oryzae*, the causal fungus of rice blast disease, negatively impacts global rice production. Wild rice (*Oryza rufipogon*), a relative of cultivated rice (*O. sativa*), possesses unique attributes that enable it to resist pathogen invasion. Although wild rice represents a major resource for disease resistance, relative to current cultivated rice varieties, no prior studies have compared the immune and transcriptional responses in the roots of wild and cultivated rice to *M. oryzae*.

**Results:**

In this study, we showed that *M. oryzae* could act as a typical root-infecting pathogen in rice, in addition to its common infection of leaves, and wild rice roots were more resistant to *M. oryzae* than cultivated rice roots. Next, we compared the differential responses of wild and cultivated rice roots to *M. oryzae* using RNA-sequencing (RNA-seq) to unravel the molecular mechanisms underlying the enhanced resistance of the wild rice roots*.* Results indicated that both common and genotype-specific mechanisms exist in both wild and cultivated rice that are associated with resistance to *M. oryzae.* In wild rice, resistance mechanisms were associated with lipid metabolism, WRKY transcription factors, chitinase activities, jasmonic acid, ethylene, lignin, and phenylpropanoid and diterpenoid metabolism; while the pathogen responses in cultivated rice were mainly associated with phenylpropanoid, flavone and wax metabolism. Although modulations in primary metabolism and phenylpropanoid synthesis were common to both cultivated and wild rice, the modulation of secondary metabolism related to phenylpropanoid synthesis was associated with lignin synthesis in wild rice and flavone synthesis in cultivated rice. Interestingly, while the expression of fatty acid and starch metabolism-related genes was altered in both wild and cultivated rice in response to the pathogen, changes in lipid acid synthesis and lipid acid degradation were dominant in cultivated and wild rice, respectively.

**Conclusions:**

The response mechanisms to *M. oryzae* were more complex in wild rice than what was observed in cultivated rice. Therefore, this study may have practical implications for controlling *M. oryzae* in rice plantings and will provide useful information for incorporating and assessing disease resistance to *M. oryzae* in rice breeding programs.

**Electronic supplementary material:**

The online version of this article (10.1186/s12284-018-0211-8) contains supplementary material, which is available to authorized users.

## Background

Rice (*Oryza sativa*) is the main food staple for approximately half of the world’s population; thus breeding for yield improvement to feed an ever-increasing world population is a critical goal (Hua et al. 2015). Wild rice (*Oryza rufipogon*), a relative of cultivated rice, possesses several unique attributes; including disease and lodging resistance, as well as drought tolerance (Ji et al. 2016; Kim et al. 2016). Unfortunately, several yield- and stress resistance-related traits present in wild rice progenitors were lost during the domestication of cultivated rice varieties (Zhang et al. 2017). Therefore, the genetic diversity of wild rice is utilized in current rice breeding efforts to recover important traits, such as disease resistance (Sheng et al. 2017). In order to preserve genetic diversity for rice breeding efforts, China has protected several conservation areas for maintaining the production of wild rice and provides research material for investigating the response of wild rice and cultivated varieties of rice to various types of biotic and abiotic stresses (Tian et al. 2017; Zhang et al. 2016a, 2016b; Zhou et al. 2016).

RNA-sequencing (RNA-seq) and microarray transcriptome analyses provide good overviews of the genetic response and inferred plant biochemical changes that occur in response to a wide range of factors (Tran and Mochida 2010; Mochida and Shinozaki 2011; Donofrio et al. 2014; Nguyen et al. 2016; Wang et al. 2016; Zhou et al. 2016; Chen et al. 2017; Nasr Esfahani et al. 2017). Transcriptomic profiles can also provide a comparison of enriched genes in two different genotypes by conducting a pairwise analysis of gene expression, and further investigation of the metabolic pathways or biological processes that are enriched in the compared genotypes (Ueno et al. 2015; Wu et al. 2015). Recently, RNA-seq analysis has been widely used to elucidate the underlying molecular mechanisms of plant stress resistance and the crosstalk that occurs between different signaling pathways (Mochida and Shinozaki 2011; AbuQamar et al. 2016; Nasr Esfahani et al. 2017). Growing evidence indicates that macro- and micro-molecules play important beneficial roles for increasing plant stress resistance (Shah 2005; Wang et al. 2011; Fatima et al. 2016; Ekchaweng et al. 2017; Kiss et al. 2017; Ma et al. 2017). Lipid and starch macromolecules are important not only for energy storage within plants, but they can also act as signaling compounds in biotic stress induced signal transduction pathways (Shah 2005; Fatima et al. 2016). Thus, it is essential to understand the connection between macromolecular substances and the resistance to both biotic and abiotic stress in plants.

*Magnaporthe oryzae*, the spontaneous fungal agent of rice blast disease, is widely distributed and causes serious reductions in rice yields worldwide (Osés-Ruiz et al. 2016; Yan and Talbot 2016). *M. oryzae* infection is most commonly initiated in rice leaves by the germination of spores and the development of appressoria, which then allow the pathogen to invade the leaves (Li et al. 2014; Foster et al. 2016). A number of studies, however, have reported that *M. oryzae* can also infect roots without the formation of appressoria (Sesma and Osbourn 2004; Marcel et al. 2010; Tucker et al. 2010). Previous studies have confirmed that some pathogenesis-related hormones, including jasmonic acid (JA), cytokinins (CKs), abscisic acid (ABA), salicylic acid (SA) and ethylene (ET), are involved in the immunity responses of rice to *M. oryzae* (Yang et al. 2013; Muller and Munne-Bosch 2015; Cao et al. 2016; Nasir et al. 2017). Although it has been reported that wild rice represents a major resource for disease resistance, relative to current cultivated varieties of rice, no studies have compared the immune responses of wild and cultivated roots of rice to *M. oryzae*. Therefore, it is essential to investigate the mechanisms associated with the resistance responses of roots of wild rice to *M. oryzae* in order to provide current practical strategies for breeding resistance to this pathogen in rice. In the current study, the transcriptomic changes of wild and cultivated rice in response to *M. oryzae* was compared using RNA-seq analysis, followed by gene enrichment and pathway analyses. The transcriptomes of inoculated and non-inoculated wild and cultivated rice plants were compared within and between the different rice species. Results from these analyses will be helpful for developing practical breeding strategies aimed at providing new varieties with improved disease resistance to *M. oryzae.*

## Methods

### Plant materials and experimental design

Seedlings of cultivated rice (*Oryza sativa* L. ssp. Japonica), Dongdao-4 (a widely grown Japonica-type cultivar in the Songnen Plain of Northeast China) (Lv et al. 2015; Zhang et al. 2016a, 2016c), and Dongxiang wild rice (a Chinese common wild rice; *Oryza rufipogon* Griff.) (Zhang et al. 2016b) were used in the current study. *Magnaporthe oryzae* Guy 11, which is well-known for its compatible interaction with the roots of *Oryza sativa* (Sesma and Osbourn 2004; Marcel et al. 2010), was used as the model pathogen strain. To establish seedling growth, cultivated and wild rice seeds were treated in 1% sodium hypochlorite for 10 min, followed by several washes with sterilized water. Seeds were then placed in petri dishes on wet filter paper, and cultured in the dark for germination. After 3 d, the germinated seeds were transplanted into pots containing autoclaved soil (4 seeds in each pot), and the pots were maintained in a growth chamber that was preset to 16 h light/8 h dark photoperiod, 26–28°C and 65% relative humidity. Soil organic matter, total nitrogen, available-nitrogen, −phosphorus, and -potassium were 31.2 g/kg, 651.92 mg/kg, 109.20 mg/kg, 7.48 mg/kg and 88.66 mg/kg, respectively. The pH of the soil was 6.31. After 10 d of growth in the chamber, when second true leaves emerged, a subset of the seedlings was inoculated with *M. oryzae* onto roots using fungal hyphae that were cultured on potato-dextrose agar. Specifically, hyphae were collected by flooding the culture plates with sterilized water; which was then poured into a subset of pots containing either cultivated or wild rice seedlings. Seven days after inoculation, disease symptoms on the roots were clearly evident. At this time, roots with infection symptoms were selected, and the middle parts of these roots (together with infected symptoms) were cut and collected for RNA extraction and microscopic analysis. For a control, the middle parts of the corresponding roots were cut and collected from the non-inoculated seedlings. Each treatment had 3 biological replicates.

### Microscopic observations of roots

After harvesting, roots were gently cleaned in tap water and subjected to phenotypic observations. Hand-cut cross-sections of roots were made and stained with a safranin-aniline blue method (Stanfield et al. 2017) and observed under a light microscope (XDS-2BI, China).

### Determination of chitinase activity, soluble sugar content and proline content

The chitinase activity in root samples was determined as described by Van Loon (Van Loon and Van Strien 1999). Soluble sugar and proline contents in roots were measured by anthrone colorimetry (Liu et al. 2015b, 2015a) and ninhydrin colorimetry (Liu et al. 2015b, 2015a), respectively.

### RNA extraction, cDNA library construction and RNA-seq analysis

RNA was extracted from three biological repeats of roots collected from each treatment using a Promega RNA extraction kit (Promega, China, LS1040) according to the manufacturer’s instructions. The quality and concentration of extracted RNA samples were assessed spectrophotometrically using a NanoDrop (NanoDrop 2000, Germany). Subsequently, cDNA libraries were constructed according to Chen et al. (2017). Paired-end sequencing (2 × 100 bp) was carried out using the Illumina HiSeq X Ten platform (Illumina, San Diego CA, USA) at the Beijing Ori-Gene Science and Technology Co., Ltd. (Beijing, China). FastaQC (http://www.bioinformatics.babraham.ac.uk/projects/fastqc/) and cutadapt (http://cutadapt.readthedocs.io/en/stable/) were used to control sequence quality. The filtered reads (~ 28 million) were mapped onto the reference genome using bowtie with default settings (http://bowtie-bio.sourceforge.net/index.shtml). Comparative analysis of gene expression was used to evaluate DEGs. Cufflinks (http://sihua.us/Cufflinks.htm) was used to conduct a *t*-test (*P* < 0.05) and identify genes that were differentially expressed in the inoculated vs non-inoculated roots of cultivated and wild rice. A false discovery rate (FDR) of 5% (*q*-value < 0.05) was used to identify highly expressed transcripts with at least a 2-fold change. GO annotations were performed using Blast2GO v2.5 based on the non-redundant (Nr) protein sequences (NCBI) and Pfam (NCBI, non-redundant nucleotide sequences) annotations, with 20,693 genes with known functions being included in GO annotation and enrichment analyses. KAAS (KEGG Automatic Annotation Server: http://www.genome.jp/kegg/kaas/) was used for the KEGG annotations.

### Quantitative reverse transcription-PCR (qRT-PCR) analysis

In order to validate the DEGs identified in the RNA-seq analysis, qRT-PCR analysis was conducted on a variety of selected genes. A Superscript III Reverse Transcriptase kit was used to generate the cDNA from the same RNA extracted for the RNA-seq analysis, and qRT-PCR was conducted using an Agilent MX3000P (Agilent, USA) with the following programmed conditions: 94 °C for 2 min; 40 cycles of 94 °C for 20 s, 57 °C for 20 s, and 72 °C for 30 s. The reaction mixture was 15 μl comprised of 1 μl cDNA, 1 μl forward primer, 1 μl backward primer, 7.5 ml SYBR Green Master Mix (2×), and 4.5 μl sterilized water. The relative expression levels of target genes were calculated using the 2^−ΔΔCq^ method. The *O. sativa β-tubline* gene was used as a reference gene in the data analysis and the utilized primers are listed in Table 1.

**Table 1:** Genes and primers used for verifying gene expression

Gene ID	Primer name	Sequence (5′- > 3′)
*Os06g0726200*	OS06G0726200-949F	CCGACCGGATTGGGTTCTAC
	OS06G0726200-1126R	AGCCATTGTGGGCATTACTGA
*Os12g0168700*	OS12G0168700-117F	TCTGCACTCAAGCCAACACT
	OS12G0168700-234R	CCAACTTCCATTGACTGCGG
*Os03g0290300*	OS03G0290300-209F	TTGAGGTTCACCATGCCGTT
	OS03G0290300-324R	CCAGCCAGGATGCAGTTGAT
*Os08g0448000*	OS08G0448000-28F	GTAGTTGTCATCACGCGCAC
	OS08G0448000-121R	GAGCGGAAGACGAACTGCTC
*Os04g0229100*	OS04G0229100-830F	CCAGAAGCAGATGCAGGCTA
	OS04G0229100-978R	ACTCACCGTCCTCTTACCGA
*Os02g0627100*	OS02G0627100-2014F	GTATCCGCTCTACCGGTTCG
	OS02G0627100-2197R	GCCTCCACACTCCACTGTTAT
*Os04g0483500*	OS04G0483500-840F	CGTCCATCAAGAAGGCGTCC
	OS04G0483500-934R	GCGGAGATGAGGAACCACAG
*Tubulin*	Tubulin-F	TACCGTGCCCTTACTGTTCC
	Tubulin-R	CGGTGGAATGTCACAGACAC

### Statistical analysis

General linear model analysis of variance was conducted to determine the impact of rice genotype and the pathogen on chitinase activity and contents of soluble sugars and proline using the SPSS 19.0 software. Heat map analysis was carried out by the pheatmap function in R 3.2.1 with the pheatmap package. Venn diagrams were generated using the VennDiagram function in R 3.2.1 with the limma package. Principal component analysis (PCA) was conducted using the PCA function in R 3.2.1 with the FactoMineR package. MapMan analysis was conducted using MapMan 3.6.0 (http://mapman.gabipd.org/web/guest) software.

## Results

### Phenotypic analysis of cultivated and wild rice roots with or without *M. oryzae* infection

**Fig. 1:**
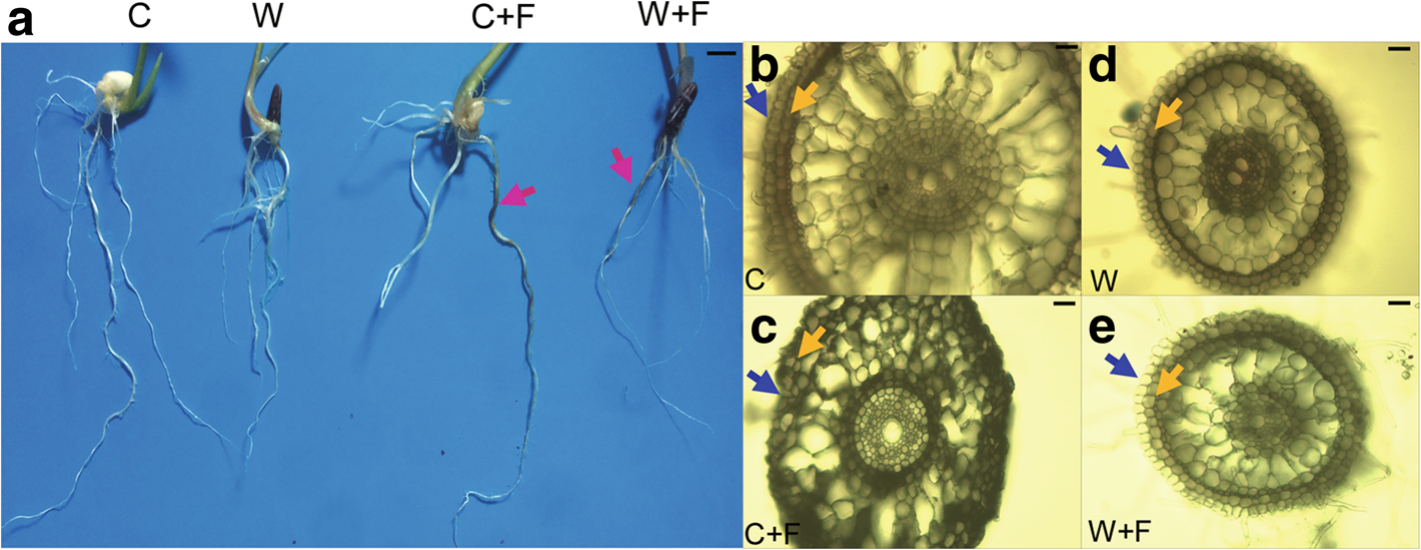
Phenotype of non-inoculated and inoculated roots of cultivated and wild rice varieties. **a** Visible phenotype of roots of C, C + F, W and W + F groups. **b**, **c**, **d** and **e** Microscopic observation of safranin-stained roots of C (**b**), C + F (**c**), W (**d**) and W + F (**e**), respectively. The four treatments were non-inoculated cultivated rice (C), cultivated rice inoculated with *Magnaporthe oryzae* (C + F), non-inoculated wild rice (W), and wild rice inoculated with *M. oryzae* (W + F). Black lines are the scale bars, which represent 2 cm in (**a**) and 5 μm in (**b**, **c**, **d**, and **e**). Pink arrows indicate the representative parts of infected roots. Blue arrows indicate the plant epidermal regions, and orange arrows indicate the cortex regions

The observed root phenotypes indicated that both cultivated and wild rice varieties developed black, decayed areas in inoculated roots that were not present in non-inoculated roots (Fig. 1a). In response to the pathogen, microscopic observations of root cross-sections demonstrated that epidermal and cortical layers were significantly more intact in wild rice than what was observed in cultivated rice roots (Fig. 1b-e). Taken together, these data suggest that wild rice roots possess a better defense against *M. oryzae* infection than cultivated rice roots. In wild rice roots, the epidermal and cortical cells inoculated with pathogen (W + F) did not exhibit significant damage in comparison to wild non-inoculated (W) rice roots (Fig. 1d, e). In contrast, significant cytomorphosis in the epidermal and cortical cells were observed between the roots of inoculated cultivated rice (C + F) and non-inoculated cultivated rice (C) (Fig. 1b, c). However, no significant phenotypic differences were observed in the aerial parts of cultivated and wild rice varieties with or without *M. oryzae* infection (Additional file 1: Figure S1).

### Comparison of stress-related indices of cultivated and wild rice roots with or without *M. oryzae* infection

**Fig. 2:**
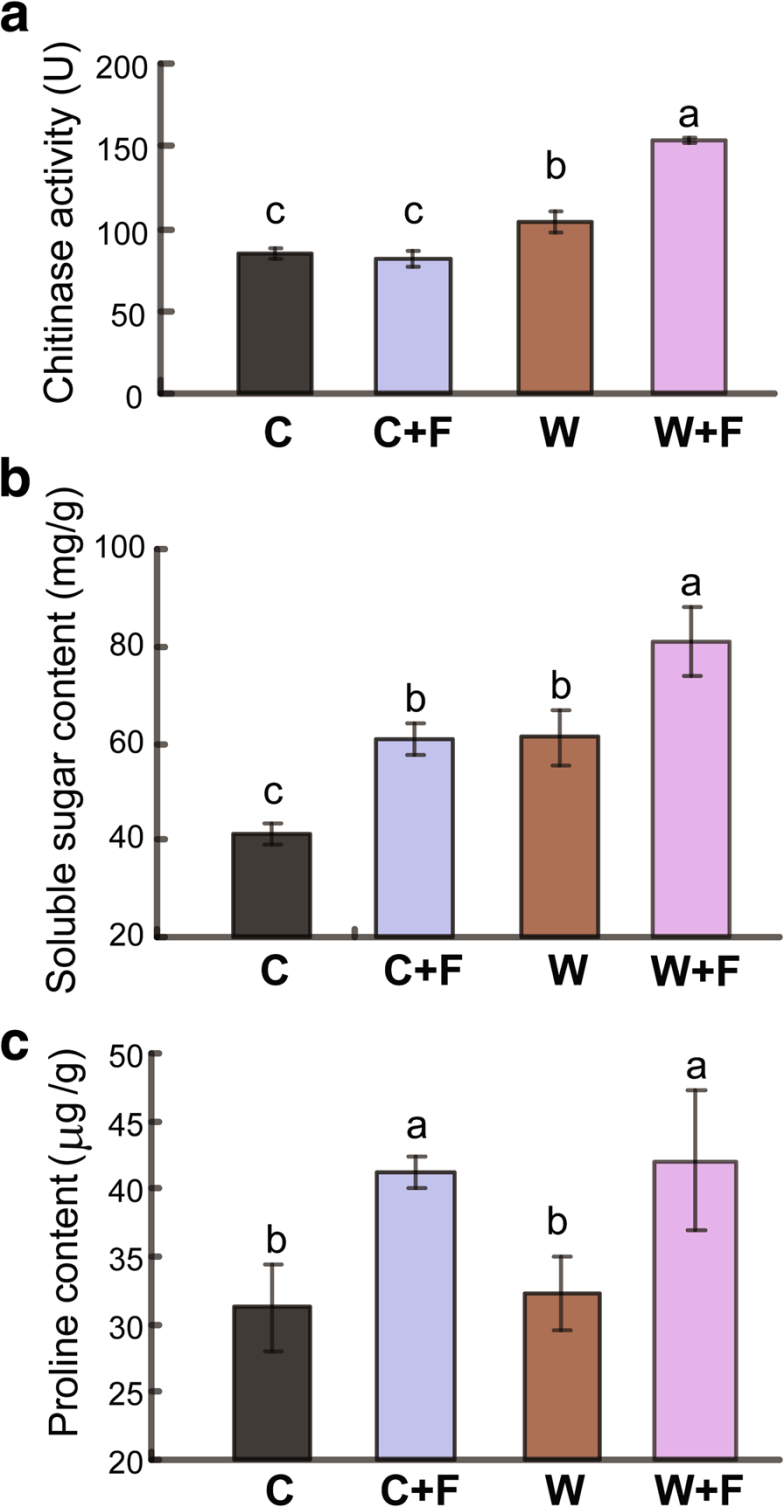
Chitinase activity, and contents of soluble sugars and proline in wild and cultivated rice roots with and without *Magnaporthe oryzae* infection. **a** Chitinase activity. **b** Soluble sugar content. **c** Proline content. The four treatments were non-inoculated cultivated rice (C), cultivated rice inoculated with *M. oryzae* (C + F), non-inoculated wild rice (W), and wild rice inoculated with *M. oryzae* (W + F). The error bars represent standard deviations of the means. Different letters above the bars indicate significant differences among samples at *P* < 0.05

Chitinase activity, soluble sugar content and proline content, which have widely been used as stress-related indices (Li et al. 2013; Sytwala et al. 2015), were analyzed in both non-inoculated and inoculated roots of wild and cultivated rice varieties. Chitinase activity was significantly higher in inoculated wild rice (153.67 U) than in non-inoculated wild rice (104.24 U; Fig. 2a). In contrast, no significant difference in the level of chitinase activity was observed between the non-inoculated and inoculated cultivated rice varieties (Fig. 2a). Both soluble sugar content and proline content were significantly higher in inoculated than in non-inoculated wild and cultivated rice plants (Fig. 2b, c). Specifically, soluble sugar content was 81.11 mg/g in the W + F group, which showed an increase by 32.47% compared with that in the W group (Fig. 2b); while it was 60.90 mg/g in the C + F group, exhibiting an increase by 47.68% relative to that in the C group (Fig. 2b). Proline content was 42.11 μg/g in the W + F group, showing a 30.65% increase compared with that in the W group (Fig. 2c); while it was 41.20 μg/g in the C + F group, displaying a 31.88% increase over that in the C group (Fig. 2c).

### Comparative genome-wide transcriptome analysis of cultivated and wild rice roots with or without *M. oryzae* infection using RNA-seq

The root transcriptomes of wild and cultivated rice varieties treated and untreated with *M. oryzae* were compared in order to elucidate their molecular responses to infection by the rice blast fungus. A total of ~ 624 million raw reads were obtained from the 12 samples, and each sample had 30.54–64.75 million raw reads (Additional file 2: Table S1). After filtering out low-quality reads, a total of ~ 556 million clean reads were obtained with an average of 84.18% that could be mapped to the rice reference genome. The percentage of clean reads (144.4–146 bp average length) from each sample that could be mapped ranged from 74.76 to 89.35% (Additional file 2: Table S1).

**Fig. 3:**
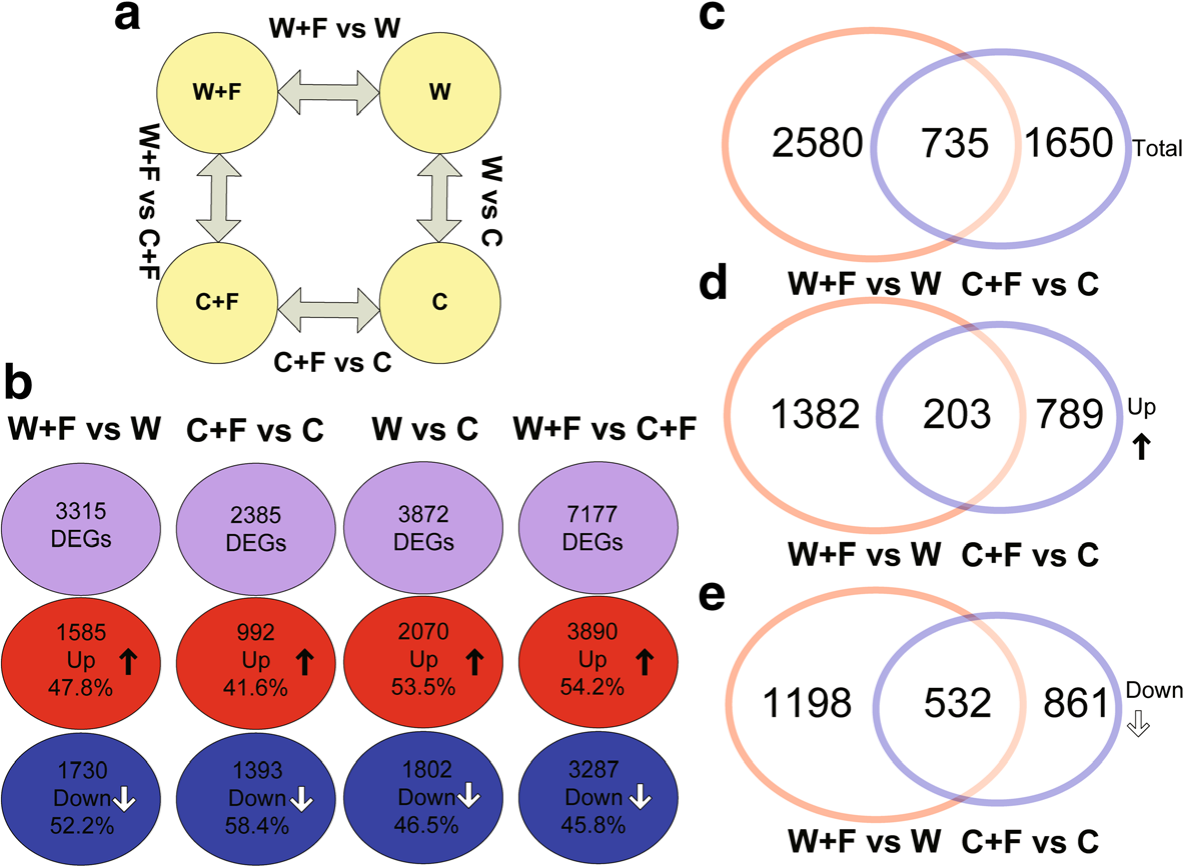
Numerical values of differentially expressed gene (DEG) analysis. (**a**) Diagrams showing the experimental design and comparisons. (**b**) Diagram showing the total, up- and down-regulated gene sets in four comparisons. (**c**, **d**, and **e**) Venn analysis of total, up- and down-regulated DEGs derived from W + F vs W and C + F vs C comparisons. The four treatments were non-inoculated cultivated rice (C), cultivated rice inoculated with *Magnaporthe oryzae* (C + F), non-inoculated wild rice (W), and wild rice inoculated with *M. oryzae* (W + F)

The number of mapped unigenes was 30,752, 31,226, 30,702 and 31,337 for the C, W, C + F and W + F samples, respectively (Additional file 3: Figure S2a). Principal component analysis (PCA) indicated that replicate samples within each sample group clustered together (Additional file 3: Figure S2b). A total of 3872 differentially expressed genes (DEGs) (2070 up- and 1802 down-regulated) were identified in the W vs. C comparison (Fig. 3b); while 7177 DEGs (3890 up- and 3287 down-regulated), were identified in the W + F vs C + F comparison (Fig. 3b). On the other hand, a total of 3315 DEGs (1585 up- and 1730 down- regulated), were present in the W + F vs W comparison (Fig. 3b); while 2385 DEGs (992 up- and 1393 down-regulated) were obtained from the C + F vs C comparison (Fig. 3b). As illustrated in the diagrams (Fig. 3c-e), the number of DEGs shared between the W + F vs W comparison and the C + F vs C comparison was 735, with 203 up- and 532 down-regulated DEGs.

### Confirmation of RNA-seq data using quantitative reverse transcription-PCR (qRT-PCR)

**Fig. 4:**
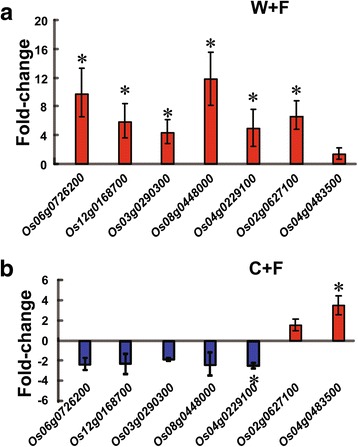
Validation of the RNA-sequencing data by quantitative reverse transcription PCR (qRT-PCR). Seven genes were selected from the RNA-sequencing data for qRT-PCR. **a** Expression patterns in C and C + F groups. **b** Expression patterns in W and W + F groups. The four treatments were non-inoculated cultivated rice (C), cultivated rice inoculated with *Magnaporthe oryzae* (C + F), non-inoculated wild rice (W), and wild rice inoculated with *M. oryzae* (W + F). The error bars represent standard deviations of the means. The asterisks above the bars indicate significant differences among the samples at *P* < 0.05

In order to confirm the results obtained by RNA-seq, the expression of 7 selected genes (*Os06g0726200*, *Os12g0168700*, *Os03g0290300*, *Os08g0448000*, *Os04g0229100* and *Os02g0627100*) was analyzed by qRT-PCR and revealed that all of these genes were up-regulated in the W + F vs W comparison (Fig. 4a). In addition, the qRT-PCR analysis also indicated that *Os04g0483500* was up-regulated in the C + F vs C comparison (Fig. 4b, Additional file 4: Table S2). In general, the expression levels revealed by qRT-PCR and RNA-seq analyses were in accordance to one another.

### Gene ontology (GO) annotation and enrichment analysis

**Fig. 5:**
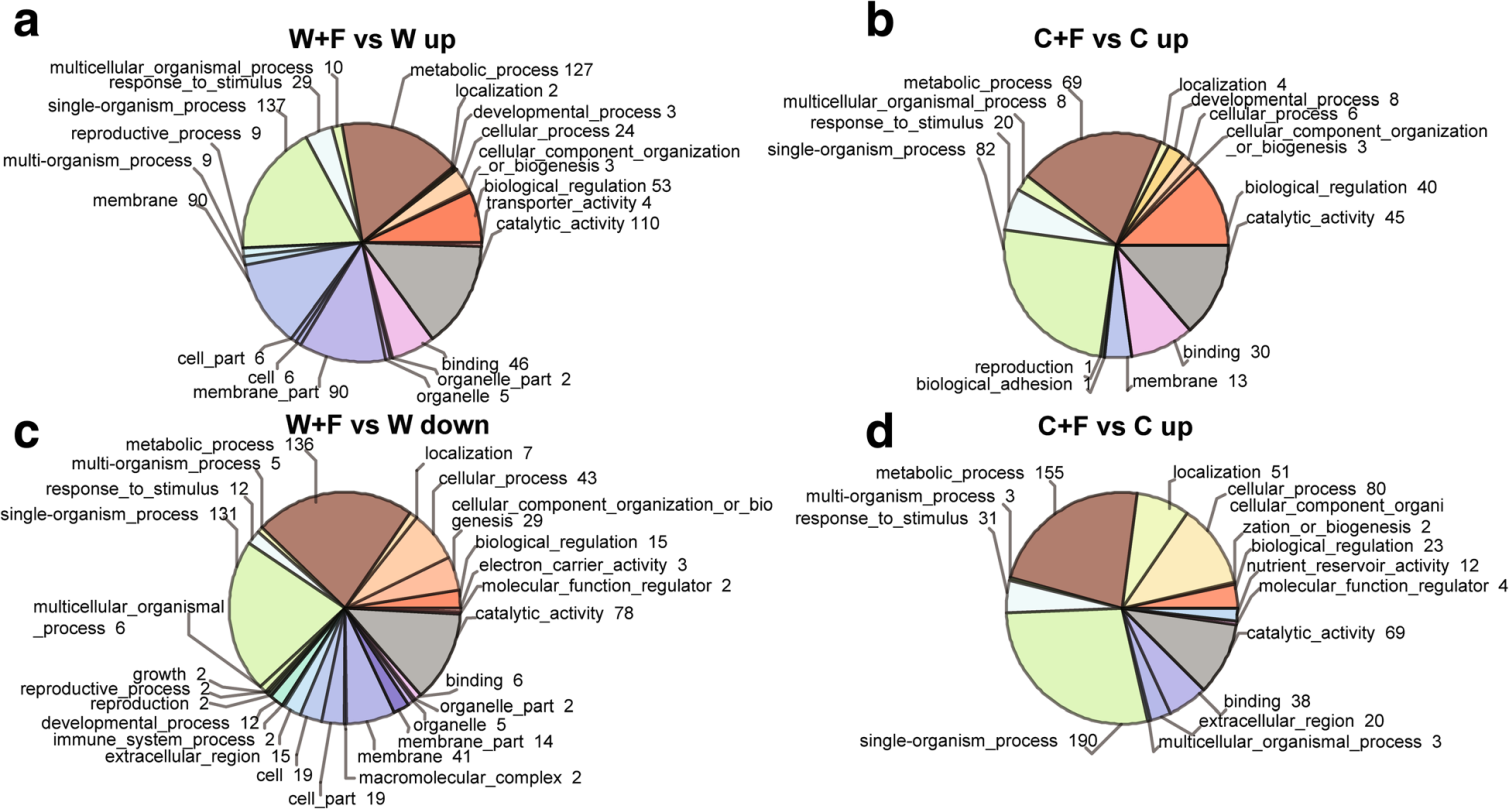
Classification of up- or down-regulated genes by gene ontology (GO) terms. **a** GO-term classification of up-regulated genes in W + F vs W comparison. **b** GO-term classification of up-regulated genes in C + F vs C comparison. **c** GO-term classification of down-regulated genes in the W + F vs W comparison. **d** GO term classification of down-regulated genes in the C + F vs C comparison. The four treatments were non-inoculated cultivated rice (C), cultivated rice inoculated with *Magnaporthe oryzae* (C + F), non-inoculated wild rice (W), and wild rice inoculated with *M. oryzae* (W + F). Numbers shown next to the terms indicate the number of up- or down-regulated genes

The DEGs of the W + F vs W and C + F vs C comparisons were first annotated using GO analysis in order to assign functional terms to the identified DEGs. A greater number of annotated DEGs in the W + F vs W comparison were identified than within the C + F vs C comparison. A total of 1375 annotated DEGs (41.5% of the total DEGs in the W + F vs W comparison), with 765 up- and 610 down-regulated genes, were identified in the W + F vs W comparison; while 1008 annotated DEGs (42.2% of the total DEGs in the C + F vs C comparison), with 330 up- and 678 down-regulated genes, were obtained from the C + F vs C comparison (Additional file 5: Figure S3). Furthermore, the GO enrichment analysis revealed that genes related to the terms ‘cell’, ‘cell part’, ‘membrane part’, and ‘organelle part’ were enriched within the cell component category in the W + F vs W comparison (Fig. 5). For instance, the GO terms ‘mitochondrion’ (GO:0005739), ‘cell wall thickening’ (GO:0052386), and ‘integral components of the inner membrane of mitochondria’ (GO:0031305) were enriched in the analysis of DEGs (Table 2). Among these DEGs, there were several noteworthy up-regulated genes such as *Os03g0678800*, a hexosyltransferase that is involved in fructose synthesis and cell wall thickening, and *Os03g0305600* which encodes a mitochondrial import inner membrane translocase that is involved in importing proteins across the outer and inner mitochondrial membranes into the matrix (Table 2). However, these observed enrichments in the W + F vs W comparison were not found in the C + F vs C comparison (Table 2). Furthermore, within the GO category biological process, the terms ‘transmembrane transporter activity’ (GO:0022857), and ‘fatty-acyl-CoA reductase (alcohol-forming) activity’ (GO:0080019) were enriched in the W + F vs W comparison, but not in the C + F vs C comparison (Fig. 5, Table 2). On the other hand, the GO term ‘regulation of secondary cell wall biogenesis’ (GO:2000652) was enriched in the C + F vs C comparison but not in the W + F vs W comparison, suggesting that secondary cell wall biogenesis might be enhanced in the C + F group (Table 3).

**Table 2:** Classification of differentially expressed genes derived from comparison between W + F and W groups (W + F vs W) using the second gene ontology (GO) term. The two treatments were wild rice without inoculation (W), and wild rice inoculated with *Magnaporthe oryzae* pathogen (W + F), respectively

GO category	function	Gene ID	Fold-change	*q*-value
GO:0052386	cell wall thickening	*Os03g0678800*	2.48	< 0.01
GO:0031305	integral component of mitochondrial inner membrane	*Os03g0305600*	2.48	< 0.01
		*Os03g0415500*	3.18	0.04
GO:0005739	mitochondrion	*Os01g0307686*	2.23	< 0.01
		*Os03g0287400*	2.45	0.05
		*Os09g0458700*	2.56	< 0.01
GO:0022857	transmembrane transporter activity	*Os01g0546100*	9.53	0.02
		*Os05g0106300*	2.82	< 0.01
		*Os05g0409500*	3.05	< 0.01
		*Os05g0493800*	2.67	< 0.01

**Table 3:** Classification of differentially expressed genes derived from comparison between C + F and C groups (C + F vs C) using the second gene ontology (GO) term. The two treatments were cultivated rice without inoculation (C), and cultivated rice inoculated with *Magnaporthe oryzae* pathogen (C + F)

GO catagory	function	Gene ID	Fold-change	*q*-value
GO:2000652	regulation of secondary cell wall biogenesis	*Os03g0720800*	2.32	< 0.01
		*Os11g0207600*	2.62	< 0.01
GO:0080019	fatty-acyl-CoA reductase (alcohol-forming) activity	*Os08g0298700*	10.39	< 0.01
		*Os09g0567500*	2.57	0.04
GO:0009788	negative regulation of abscisic acid-activated signaling pathway	*Os01g0884300*	2.72	< 0.01
		*Os05g0421600*	5.11	< 0.01
GO:0007155	cell adhesion	*Os09g0520800*	2.09	< 0.01

### Expression of ethylene (ET)-related and jasmonic acid (JA)-related genes

**Fig. 6:**
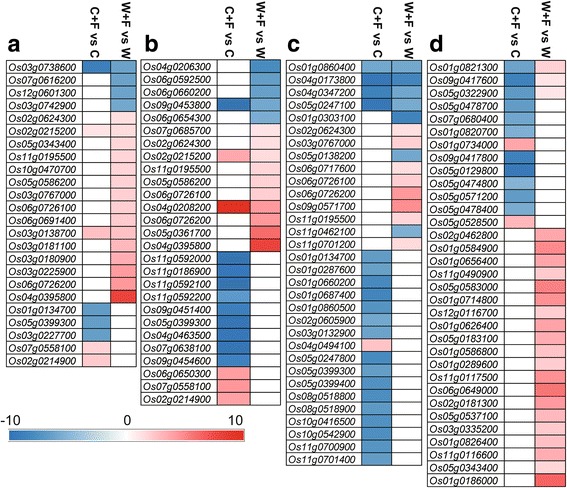
Analysis of (**a**) jasmonic acid (JA)-, (**b**) ethylene (ET)-, (**c**) chitinase-, and (**d**) WRKY- related genes that were differentially expressed in W + F vs W and C + F vs C comparisons using the fold-change values. The four treatments were non-inoculated cultivated rice (C), cultivated rice inoculated *Magnaporthe oryzae* (C + F), non-inoculated wild rice (W), and wild rice inoculated with *M. oryzae* (W + F). Color intensity indicates the fold-change values as designated by the colored bar. The white color represents unchanged gene expression

It is well known that JA and ET play critical roles in plant responses to various pathogens (Verma et al. 2016; Withers and Dong 2016; AbuQamar et al. 2017). Thus, DEGs of the W + F vs W and C + F vs C comparisons related to ET and JA were selected for GO enrichment analysis. Eight JA-related DEGs (4 up- and 4 down-regulated) were identified in the C + F vs C comparison, whereas 19 JA-related DEGs (15 up- and 4 down-regulated) were found in the W + F vs W comparison (Fig. 6a). These data suggest that, in response to *M. oryzae*, greater active modulation of JA signaling occurs in wild than cultivated varieties. For instance, the JA-related *cytochrome P450 subfamily A1* (*CYP74A1*; *Os03g0767000*), *TIFY9* (*Os04g0395800*) and *Gretchen Hagen 3.5* (*GH3.5*; *Os05g0586200*) genes were among the up-regulated DEGs in the W + F vs W comparison (Fig. 6a). On the other hand, our analysis revealed that 16 ET-related DEGs (5 up- and 11 down-regulated) were present in the C + F vs C comparison, and 15 ET-related DEGs (10 up- and 5 down-regulated) were found in the W + F vs W comparison (Fig. 6b). For example, a gene encoding an EIN3-like protein [*Os07g0685700*/*ethylene insensitive-3* (*EIN3*)], which functions in ethylene signal transduction, was identified in the W + F vs W comparison (Lee and Kim 2003).

### Chitinase- and WRKY transcription factors (TFs)-related genes

The Ensembl database and a MapMan analysis based on the Plant Proteome Database (http://ppdb.tc.cornell.edu/) were used to identify DEGs related to chitinase and WRKY TFs, respectively, within the GO annotations. The analysis identified 21 DEGs (1 up- and 20 down-regulated) related to chitinase in the C + F vs C comparison, and 15 DEGs (8 genes up- and 7 down-regulated) related to chitinase in the W + F vs W comparison. *Os03g0767000/CYP74A1*, which encodes the expression of the key enzyme (*allene oxide synthase 1*) for JA synthesis, was up-regulated in the W + F vs W comparison (Fig. 6c). Furthermore, gene *Os06g072620*0/*CHT1*, encoding chitinase 1, was also up-regulated in the W + F vs W comparison. Further analysis identified 13 DEGs (1 up- and 12 down-regulated) related to WRKY in the C + F vs C comparison, and 23 DEGs related to WRKY were all up-regulated in the W + F vs W comparison (Fig. 6d, Additional file 6: Figure S4).

### Defense-related pathway analysis

In the next line of our study, Kyoto Encyclopedia of Genes and Genomes (KEGG) annotation was performed for the DEGs in the C + F vs C and W + F vs W comparisons in order to identify DEGs related to disease defense pathways. Results indicated that phenylpropanoid synthesis-, lignin synthesis- and diterpenoid metabolism-related genes were among the DEGs identified in the W + F vs W comparison. For instance, *Os08g0448000* and *Os04g0229100*, which encode 4-coumarate:CoA ligase (*4CL*) and cinnamyl-alcohol dehydrogenase (*CAD6*), respectively, were among the up-regulated phenylpropanoid synthesis-related genes. An ω-3 fatty acid desaturase encoding gene (*Os03g0290300*) was also up-regulated in the W + F vs W comparison of DEGs (Table 4, Additional file 7: Figure S5), which is important for the synthesis of unsaturated fatty acids.

**Table 4:** Kyoto Encyclopedia of Genes and Genomes pathway analysis of up-regulated differentially expressed genes derived from comparison W + F versus W groups (W + F vs W) and comparison C + F versus C groups (C + F vs C). The four treatments were non-inoculated cultivated rice (C), cultivated rice inoculated with *Magnaporthe oryzae* (C + F), non-inoculated wild rice (W), and wild rice inoculated with *M. oryzae* (W + F)

Pathways	Gene ID	Description	W + F vs W Fold-change	*q*-value	C + F vs C Fold-change	*q*-value
Fatty acid degradation	*Os02g0647900*	alcohol dehydrogenase	5.70	0.02	unchanged	1.00
	*Os02g0730000*	aldehyde dehydrogenase (NAD+)	2.46	< 0.01	unchanged	0.19
	*Os11g0210600*	alcohol dehydrogenase	9.17	< 0.01	unchanged	0.99
	*Os12g0168700*	long-chain acyl-CoA synthetase	2.29	0.04	unchanged	0.06
Biosynthesis of unsaturated fatty acids	*Os03g0290300*	omega-3 fatty acid desaturase (delta-15 desaturase)	2.41	< 0.01	unchanged	0.77
	*Os07g0416900*	omega-6 fatty acid desaturase (delta-12 desaturase)	2.54	< 0.01	−3.16	0.02
	*Os07g0417200*	omega-6 fatty acid desaturase (delta-12 desaturase)	2.43	< 0.01	−4.12	< 0.01
Starch and sucrose metabolism	*Os01g0311800*	pectinesterase	4.30	< 0.01	−2.98	< 0.01
	*Os05g0580000*	glucose-1-phosphate adenylyltransferase	2.11	< 0.01	unchanged	0.08
	*Os07g0607400*	pectinesterase	3.50	< 0.01	unchanged	0.02
	*Os08g0445700*	trehalose 6-phosphate synthase/phosphatase	2.28	< 0.01	unchanged	0.85
	*Os09g0298200*	glucose-1-phosphate adenylyltransferase	2.92	< 0.01	unchanged	0.15
	*Os09g0504000*	UDP-glucuronate 4-epimerase	2.49	< 0.01	unchanged	0.75
Diterpenoid biosynthesis	*Os01g0757200*	gibberellin 2-oxidase	4.41	< 0.01	unchanged	0.30
	*Os02g0569900*	ent-cassa-12,15-diene 11-hydroxylase	3.31	< 0.01	unchanged	< 0.01
	*Os04g0179200*	momilactone-A synthase	2.25	< 0.01		< 0.01
Phenylpropanoid biosynthesis	*Os01g0963000*	peroxidase	3.75	< 0.01	−2.62	< 0.01
	*Os02g0467600*	trans-cinnamate 4-monooxygenase	4.59	< 0.01	unchanged	0.25
	*Os02g0627100*	phenylalanine ammonia-lyase	2.51	< 0.01	unchanged	0.04
	*Os04g0518400*	phenylalanine ammonia-lyase	4.64	< 0.01	−3.02	< 0.01
	*Os04g0651000*	peroxidase	2.76	< 0.01	unchanged	< 0.01
	*Os07g0677100*	peroxidase	5.04	< 0.01	unchanged	0.03
	*Os09g0127300*	cinnamoyl-CoA reductase	2.25	< 0.01	2.172611	0.000
	*Os10g0109600*	peroxidase	2.03	< 0.01	unchanged	0.401
	*Os11g0112200*	peroxidase	6.48	< 0.01	unchanged	0.033
Lignin	*Os04g0518400*	phenylalanine ammonia-lyase	4.64	< 0.01	−3.02	< 0.01
	*Os02g0626600*	phenylalanine ammonia-lyase	2.83	< 0.01	unchanged	0.45
	*Os02g0627100*	phenylalanine ammonia-lyase	2.51	< 0.01	unchanged	0.04
	*Os08g0448000*	4-coumarate--CoA ligase	2.55	< 0.01	unchanged	0.96
	*Os11g0643100*	Tryptamine benzoyltransferase	7.25	< 0.01	unchanged	0.65
	*Os04g0229100*	cinnamyl alcohol dehydrogenase (CAD6)	2.01	< 0.01	unchanged	< 0.01
Fatty acid elongation	*Os03g0245700*	3-ketoacyl-CoA synthase	unchanged	0.27	3.77	< 0.01
	*Os04g0483500*	17beta-estradiol 17-dehydrogenase/very-long-chain 3-oxoacyl-CoA reductase	unchanged	0.45	3.13	< 0.01
	*Os05g0574600*	3-ketoacyl-CoA synthase	unchanged	0.04	3.54	< 0.01
	*Os11g0591200*	3-ketoacyl-CoA synthase	unchanged	0.76	3.27	< 0.01
Wax and cutin synthesis	*Os01g0854800*	fatty acid omega-hydroxylase	unchanged	0.04	2.23	< 0.01
	*Os02g0666500*	fatty acid omega-hydroxylase	unchanged	0.41	3.86	< 0.01
	*Os04g0560100*	fatty acid omega-hydroxylase	unchanged	0.46	2.62	< 0.01
	*Os09g0567500*	fatty acyl-CoA reductase	unchanged	0.87	2.57	0.048
Peroxisome	*Os03g0583800*	protein Mpv17	2.18	< 0.01	2.30	< 0.01
	*Os08g0502700*	alanine-glyoxylate transaminase/serine-glyoxylate transaminase/serine-pyruvate transaminase	3.148	< 0.01	2.03	0.04
	*Os09g0567500*	fatty acyl-CoA reductase	unchanged	0.87	2.57	0.04
Flavone and flavonol biosynthesis	*Os10g0320100*	flavonoid 3′-monooxygenase	unchanged	0.36	2.26	0.04

The DEGs in the C + F vs C comparison included enriched genes that were related to fatty acid elongation, wax and cutin syntheses, phenylpropanoid metabolism, and flavone synthesis. For example, the up-regulated DEGs included genes encoding 3-ketoacyl-CoA synthase (*Os03g0245700*), fatty acid ω-hydroxylase (*Os01g0854800*), and flavonoid 3′-monooxygenase (*Os10g0320100*) (Table 4).

## Discussion

### Phenotype of cultivated and wild rice roots infected with *M. oryzae*

*M. oryzae* is a well-known leaf pathogen of rice and its leaf infection process has been well characterized. A study by Sesma and Osbourn (2004) changed the scientific perception of this pathogen with the observation that *M. oryzae* could also infect rice roots; resulting in necrosis, root loss and yield reduction. In the present study, we demonstrated that similar lesions and browning occurred in both cultivated (*Oryza sativa*) and wild (*Oryza rufipogon)* rice after the inoculation of roots with *M. oryzae* (Fig. 1a). After inoculation with the pathogen, microscopic observations of root cross-sections revealed that epidermal and cortical cells were more intact in wild rice than cultivated rice (Fig. 1b). Epidermal and cortical cells play an essential role in plant disease resistance (Ma and Yamaji 2006). Additionally, chitinase can serve as a defense-related enzyme that inhibits fungal growth due to its function in breaking down chitin (Sytwala et al. 2015). Proline and soluble sugar contents also play an important role in both biotic and abiotic stress resistance in plants (Li et al. 2013; Liu et al. 2014; Mostofa et al. 2017). These parameters were induced in infected roots of both wild and cultivated rice in comparison to the respective uninfected control (Fig. 2b, c); suggesting their involvement in defense against *M. oryzae* in rice. Furthermore, our analyses of chitinase activity and soluble sugar content indicated that these two biochemical components were significantly higher in wild rice roots than in cultivated rice roots during *M. oryzae* infection (Fig. 2); indicating that they may play more essential roles in the defense response of wild rice roots to *M. oryzae* than in cultivated rice.

### Transcriptome and GO enrichment analyses

In both cultivated and wild rice, we obtained a large, high-quality transcriptome dataset of non-inoculated and inoculated roots with the rice blast fungus, *M. oryzae* (Additional file 2: Table S1). DEGs were identified for inoculated vs non-inoculated roots of cultivated rice (C + F vs C) and wild rice (W + F vs W) (Fig. 3). The number of DEGs was higher in the W + F vs W comparison than in the C + F vs C comparison (Fig. 3), which may indicate that wild rice has a more complex response to *M. oryzae* than cultivated rice.

GO analysis indicated that the total DEGs identified in W + F vs W were enriched in the GO terms ‘cell’, ‘cell part’, ‘membrane part’, ‘organelle’ and ‘organelle part’ (Fig. 5). Furthermore, the term ‘cell wall thickening’ was more highly enriched in wild rice than in cultivated rice in response to inoculation with the pathogen (Fig. 5, Table 2); potentially indicating that cell walls might thicken as part of the defense response in wild rice compared with the cultivated one. In addition, in wild rice, the enrichment of up-regulated genes in the terms ‘integral component of mitochondrial inner membrane and mitochondria’ (Fig. 5, Table 2) suggests that energy production and consumption increased in response to the presence of the pathogen (Berkowitz et al. 2016). Within the category biological process, the term ‘transmembrane transporter activity’ was enriched (Table 2) with up-regulated genes, suggesting that macro- or micro-molecules were more highly transported through membranes in response to the pathogen in wild rice. However, these GO enrichments were not found in cultivated rice, demonstrating differential responses of wild and cultivated varieties to *M. oryzae* infection. The term ‘biological adhesion’ was enriched in the C + F vs C comparison (Table 3), but not in the W + F vs W comparison, which might be due to the immune response of cultivated rice to the invading fungal hyphae of the pathogen (Hong et al. 2016). In addition, *Os11g0207600*, which encodes a Myb-like protein and was classified into GO terms of ‘regulation of secondary cell wall biogenesis’, was also enriched in the C + F vs C comparison but not in the W + F vs W comparison; suggesting that *M. oryzae* might activate the function related to secondary cell wall synthesis in cultivated rice in response to the infection.

### Defense signaling and related proteins

JA, ET, and chitinase activity have been reported to play an important role in disease resistance responses in rice plants (Richa et al. 2016). In the present study, results based on the GO analysis revealed that up-regulated genes related to JA and ET synthesis were more enriched in the W + F vs W comparison than in the C + F vs C comparison (Fig. 6a, b); indicating that JA and ET were involved in the resistance response of wild rice to *M. oryzae*. Interestingly, JA and ET are involved in the induced systemic resistance (ISR) in plants (Pangesti et al. 2016), suggesting that ISR plays an important role in the response of wild rice roots to the pathogen. Several studies have reported that ET can improve the JA-regulating system (Zhang et al. 2007; Caarls et al. 2017). In this regard, *GH3.5/Os05g0586200*, which encodes the jasmonic acid-amido synthetase (*JAR1*) that plays an important positive regulatory role in JA- and ET-dependent ISR response in plants (Chen et al. 2009), was up-regulated in the W + F vs W comparison (Fig. 6a, b). Additionally, more up-regulated genes controlling chitinase biosynthesis (e.g. *CHT3* and *CHT1*) and *WRKY* TFs (e.g. *OS06G0649000* and *OS05G0537100*) were enriched in the W + F vs W comparison than in the C + F vs C comparison (Fig. 6c, d). Both chitinase and WRKY TFs are known to play an important role in plant disease resistance (Hu et al. 2012; Hwang et al. 2016), suggesting that the enhanced defense of wild rice against *M. oryzae* (Fig. 1) might be attributed to the actions of chitinase and WRKY TFs. Interestingly, WRKY TFs also regulate some aspects of secondary metabolism, such as lignin, phenylpropanoid and diterpenoid synthesis (Schluttenhofer and Yuan 2015). Schluttenhofer et al. (2014) reported that 80% of WRKY TFs are associated with and reflect the activation of JA signaling pathways. In our study, genes encoding WRKY TFs were significantly up-regulated in the W + F vs W comparison (Fig. 6c, d). Among them, WRKY53 is well known among WRKY TFs for its positive regulatory role in response to plant pathogens (Hu et al. 2012). Previous studies also showed that SA, ABA and CK contents, or genes responsive to these hormones, were significantly increased in rice leaves in response to *M. oryzae* infection (Verma et al. 2016; Cao et al. 2016)*.* However, the expression levels of SA-, ABA- and CKs-responsive genes were not significantly altered in this study; suggesting that SA, ABA and CKs may not play important roles in rice roots responding to *M. oryzae.*

Thus, the transcriptome comparisons between inoculated and non-inoculated groups of cultivated and wild rice indicate that the expression of JA, ET and chitinase biosynthesis-related genes, and some WRKY TFs encoding genes, is more highly up-regulated in wild rice than in cultivated rice in response to *M. oryzae*. As a result, it is plausible that plant hormones and TFs may play essential roles in the disease resistance response.

### Analysis of defense-related primary and secondary metabolic pathways

Pathway analysis revealed that both primary and secondary metabolic pathways are significantly modulated in response to *M. oryzae* in both the C + F vs C and W + F vs W comparisons (Table 4). Enrichment of the phenylpropanoid synthesis pathway was shared in the C + F vs C and W + F vs W comparisons (Table 4). Phenylpropanoid is one of the primary metabolites that has been frequently cited for its role in plant response to pathogens (Baetz and Martinoia 2014). Our results indicate that the phenylpropanoid synthesis pathway is activated in the roots of both wild and cultivated rice in response to *M. oryzae* (Table 4)*.*

The secondary metabolites lignin and flavone are derived from phenylpropanoid (Desta et al. 2016). In the present study, the lignin synthesis pathway, as reflected by the elevated expression of 4-coumarate:CoA ligase (*4CL*) and *CAD6,* was enriched in the W + F vs W comparison (Table 4), while flavone synthesis was enriched in the C + F vs C comparison (Table 4). These results suggest that the increase in phenylpropanoid metabolism in response to the rice blast fungus may be directed toward lignin and flavone synthesis in the roots of wild and cultivated rice, respectively. Diterpenoid, a type of lipid metabolite, represents secondary metabolites associated with disease resistance in plants (Chaturvedi et al. 2012). In addition, diterpenoid also has the ability to elicit acquired systemic resistance (ASR) (Chaturvedi et al. 2012). Unlike the C + F vs C comparison, diterpenoid synthesis-related genes were enriched in the W + F vs W comparison; indicating that this metabolite may function as part of the ASR mechanism to *M. oryzae* in wild rice. The synthesis of diterpenoid requires isoprene as a precursor, and isoprene production is dependent on acetyl-CoA which is a product of either the tricarboxylic acid (TCA) cycle or fatty acid degradation (Chaturvedi et al. 2012). Diterpenoid synthesis was also found to be enhanced in rice leaves after *M. oryzae* infection (Kawahara et al. 2012). In our study, the KEGG pathway analysis indicated that DEGs related to fatty acid degradation were more enriched in the W + F vs W comparison than the C + F vs C comparison (Table 4), suggesting that fatty acid degradation might have promoted diterpenoid synthesis in wild rice during *M. oryzae* infection. Since it can provide energy and can serve as a precursor for defense-related metabolites, fatty acid degradation is an important response to pathogenic fungi (Buchanan-Wollaston et al. 2003). In accordance to these previous findings, the *Os03g0290300* gene, which encodes a ω-3 fatty acid desaturase involved in the synthesis of unsaturated fatty acids, was also up-regulated in the W + F vs W comparison but not in the C + F vs C comparison (Table 4). As previously indicated, the up-regulated genes related to JA synthesis were also more highly enriched in the W + F vs W comparison than in the C + F vs C comparison (Fig. 6). In this regard, linolenic acid is commonly known as one of the unsaturated fatty acids that serves as a precursor for JA synthesis (Goepfert and Poirier 2007). Therefore, our results may indicate that unsaturated fatty acid synthesis promotes JA synthesis in wild rice roots in response to *M. oryzae* but not in that of cultivated rice. Starch metabolism, which can also provide energy and acetyl-CoA for the shikimic acid pathway where phenylpropanoid synthesis takes place (Henkes et al. 2001; Zabalza et al. 2017), was also greater in the W + F vs W comparison than in the C + F vs C comparison (Table 4).

The activated pathways in cultivated rice were different than those observed in wild rice. Wax, cutin and flavones are secondary plant metabolites (Shah 2005), of which wax and cutin are derived from fatty acids and can make plant cell walls more resistant to invading hyphae and fungal enzymes (Lattanzio et al. 2006). Interestingly, fatty acid elongation-related genes were more highly enriched in the C + F vs C comparison than in the W + F vs W comparison (Table 4). This observation suggests that fatty acid elongation may have promoted wax and cutin syntheses in cultivated rice roots in response to *M. oryzae*. The phenylpropanoid and flavone synthesis pathways are also associated with stress resistance responses in plants (Nicholson and Hammerschmidt 1992; Besseau et al. 2007). DEGs related to peroxisome synthesis were also up-regulated in the C + F vs C comparison but not in the W + F vs W comparison (Table 4). Peroxisomes function in the elimination of reactive oxygen species (ROS) (Reumann and Bartel 2016), suggesting that the defense of cultivated rice roots against *M. oryzae* might be associated with ROS scavenging. Interestingly, several photosynthesis-related genes were down-regulated in roots (Matsumura et al. 2003), while pathogenesis-related and phytoalexin biosynthesis-related genes were up-regulated in shoots that were infected with *M. oryzae* (Kawahara et al. 2012). These data are in agreement to the differential expression patterns in roots and leaves of rice subjected to *M. oryzae* infection that were also observed by Marcel et al. (2010).

### Model of the response of cultivated and wild rice roots to *M. oryzae*

**Fig. 7:**
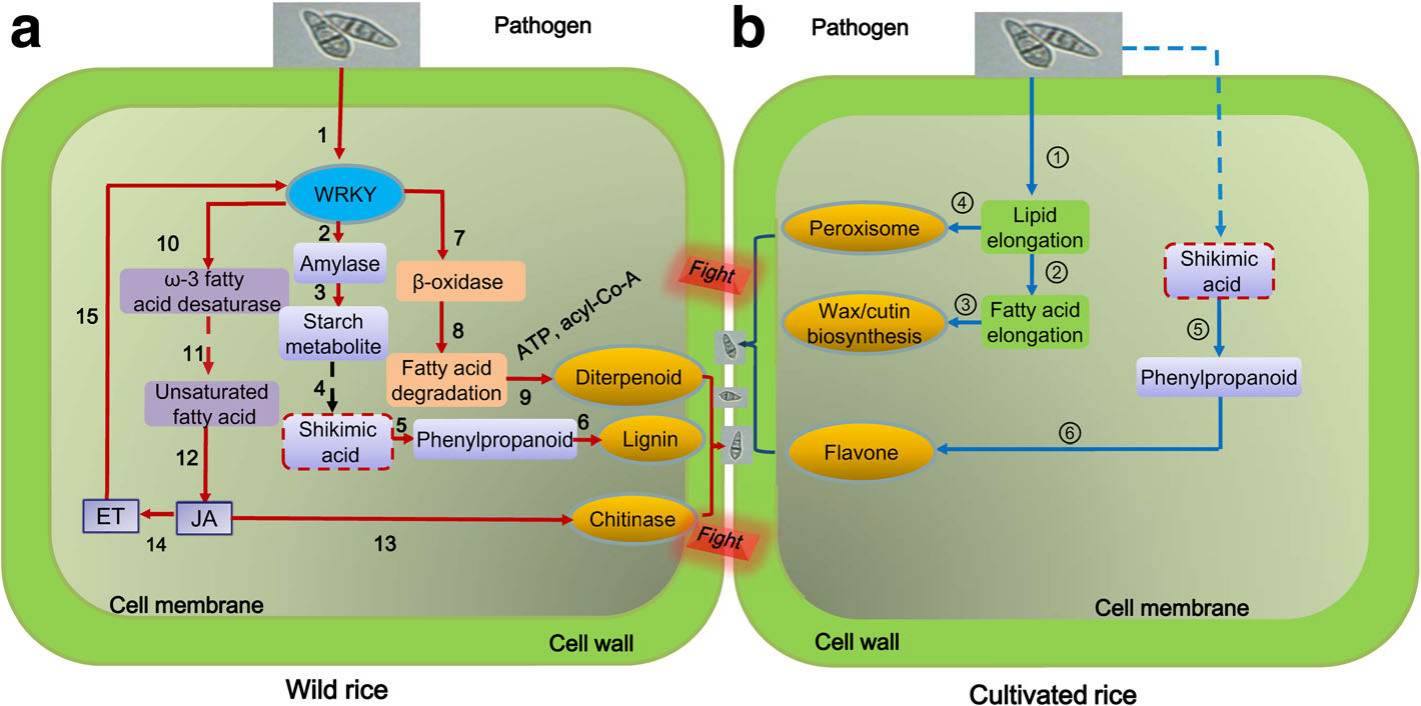
Hypothetical model of wild and cultivated rice varieties in response to pathogen attack. **a** Defense responses of wild rice roots in response to *Magnaporthe oryzae*. In the roots of wild rice, fatty acids were degraded and served as precursors for diterpenoid synthesis. Fatty acids were desaturated by an ω-3 fatty acid desaturase to produce unsaturated fatty acids that were then used to promote linolenic acid synthesis. The linolenic acid subsequently promoted jasmonic acid (JA) synthesis which then induced systemic resistance and could promote chitinase activity. Starch was metabolized to produce shikimic acid for phenylpropanoid synthesis. The phenylpropanoid was then used to produce lignin that was subsequently infused into the cell walls of roots in order to increase resistance to *M. oryzae*. **b** Defense responses of cultivated rice roots in response to *M. oryzae*. In response to *M. oryzae,* roots of cultivated rice induced genes related to elongation of fatty acids. Elongated fatty acid is then promoted synthesis of wax and cutin which are infused into cell walls of roots to promote resistance to *M. oryzae*. Phenylpropanoid metabolism was elevated in response to the pathogen, and was directed to flavone synthesis rather than lignin synthesis. Solid arrows indicate the identified pathway. Dotted arrows indicate the supposed pathways. 1. Pathogen elicits WRKY TFs (Fig. 6d); 2. WRKY TFs elicit amylase (Table 4); 3. Starch metabolite occurs (Table 4); 4. Shikimic acid pathway assumably occurs; 5. Phenylpropanoid synthesis (Table 4); 6. Lignin synthesis (Table 4, Additional file 7: Figure S5); 7. WRKY induces β-oxidase activity (Table 4); 8. Fatty acid is degraded (Table 4); 9. NADPH and acyl-CoA promote diterpenoid synthesis; 10. WRKY TFs induce ω-3 fatty acid desaturase activity (Table 4); 11. ω-3 fatty acid desaturase promotes linolenic acid synthesis [Table 4 and assumed, Simopoulos (2016)]; 12. JA synthesis occurs (Fig. 6a); 13. JA promotes chitinase activity (Fig. 6a); 14. ET synthesis is induced under stress; 15. JA and ET promote production of WRKY TFs [Fig. 6b, Schluttenhofer and Yuan 2015]; ① Fatty acid synthesis is promoted (Table 4); ② Fatty acid is accumulated (Table 4); ③ Wax and cutin synthesis [Table 4, Lattanzio et al. 2006]; ④ Peroxisome is produced (Table 4); ⑤ Phenylpropanoid metabolism occurs (Table 4); ⑥ Flavone synthesis [Table 4, Zhao et al. 2016]. The dashed arrows and boxes represent the putative pathway in accordance to published literature, while the solid lines represent the findings of the present study. Steps 4 and 11 occur in mitochondria. The germinating spores shown represent the pathogen *Magnaporthe oryzae*. ET, ethylene; WRKY TFs, WRKY transcription factors

Based on the findings of the present study, we constructed a model of the pathways involved in the resistance response of wild and cultivated rice to the rice blast fungus *M. oryzae* (Fig. 7). In wild rice, the response to *M. oryzae* involved fatty acid degradation to generate precursors that are required for diterpenoid synthesis. Fatty acids were also desaturated by an ω-3 fatty acid desaturase and were used for the synthesis of unsaturated fatty acids, which in turn promoted the synthesis of linolenic acid. Linolenic acid then promoted JA synthesis, which subsequently resulted in an ISR response. Starch was also metabolized for generating the energy needed to produce shikimic acid and to promote phenylpropanoid synthesis, which was subsequently used in lignin production. The lignin was then infused into the cell walls of root cells to increase their resistance to *M. oryzae.* An enrichment in mitochondrial activity in wild rice vs cultivated rice would assist in providing sufficient energy that is necessary for the activated defense responses. In cultivated rice, however, fatty acid elongation promoted wax and cutin synthesis for infusion into cell walls to increase resistance to *M. oryzae*. Phenylpropanoid metabolism also increased, but was channeled into the production of the secondary metabolite, flavone.

## Conclusions

The present study revealed that the primary response of roots to *M. oryzae* in wild rice is more complex and diverse than in cultivated rice. WRKY TFs, chitinase activity, JA, ET, lignin, as well as phenylpropanoid and diterpenoid synthesis, were all associated with the resistance responses displayed by the roots of wild rice to *M. oryzae*. The resistance responses in roots of cultivated rice, however, only involved genes associated with phenylpropanoid, flavones and wax. Modulation of primary metabolism (starch, soluble sugars, proline and chitinase activity), and phenylpropanoid synthesis were common responses that were shared between both cultivated and wild rice. The modulation of secondary metabolism, and the production of phenylpropanoid, were directed towards lignin synthesis in wild rice and flavone synthesis in cultivated rice, respectively. In addition, the analysis of genes associated with nutrient metabolism indicated that fatty acid and starch metabolism was modulated in both wild and cultivated rice in response to the pathogen. In this regard, however, lipid acid synthesis was specifically enriched in cultivated rice, while lipid acid degradation was specifically enriched in wild rice in response to *M. oryzae*. The results of the study may have practical implications for controlling *M. oryzae* in rice plantings and can provide useful information for incorporating and assessing disease resistance to *M. oryzae* in rice breeding programs.

## Additional files

Additional file 1: **Figure S1.** Aerial parts of non-inoculated and inoculated cultivated and wild rice varieties. The four treatments were non-inoculated cultivated rice (C), cultivated rice inoculated with *Magnaporthe oryzae* (C + F), non-inoculated wild rice (W), and wild rice inoculated with *M. oryzae* (W + F). Black line indicates the scale bar of 1 cm. (PDF 239 kb)

Additional file 2: **Table S1.** Summary of Illumina RNA-sequencing reads mapped to the reference genes. The four treatments were non-inoculated cultivated rice (C), cultivated rice inoculated with *Magnaporthe oryzae* (C + F), non-inoculated wild rice (W), and wild rice inoculated with *M. oryzae* (W + F). (PDF 7 kb)

Additional file 3: **Figure S2.** (a) Principal component analysis (PCA) of the total samples and (b) total mapped unigenes for the C, W, C + F and W + F groups. The four treatments were non-inoculated cultivated rice (C), cultivated rice inoculated with *Magnaporthe oryzae* (C + F), non-inoculated wild rice (W), and wild rice inoculated with *M. oryzae* (W + F). (PDF 32 kb)

Additional file 4: **Table S2.** RNA-sequencing and quantitative reverse transcription PCR (qRT-PCR) data of the verified genes. The fold-changes shown were obtained from RNA-sequencing and qRT-PCR data derived from C + F vs C comparison and W + F vs W comparison. Red, blue and black colors indicate the up- (fold-changes ≥2 with a *q*-value < 0.05), down-regulated (fold-changes ≤2 with a *q*-value < 0.05) and unchanged genes, respectively. The four treatments were non-inoculated cultivated rice (C), cultivated rice inoculated with *Magnaporthe oryzae* (C + F), non-inoculated wild rice (W), and wild rice inoculated with *M. oryzae* (W + F). (PDF 54 kb)

Additional file 5: **Figure S3.** (a) Total, (b) up- and (c) down-regulated differentially expressed genes (DEGs) identified in comparison W + F vs W and comparison C + F vs C by gene ontology annotation analysis. The four treatments were non-inoculated cultivated rice (C), cultivated rice inoculated with *Magnaporthe oryzae* (C + F), non-inoculated wild rice (W), and wild rice inoculated with *M. oryzae* (W + F). (PDF 15 kb)

Additional file 6: **Figure S4.** MapMan analysis of the genes and pathways responsive to the pathogenic invasion using the differentially expressed genes derived from (a) comparison C + F vs C, and (b) comparison W + F vs W. Red arrows indicate the pathways enriched in up-regulated genes. Blue and red colors indicate down- and up-regulated genes, respectively. The colored bar in each panel shows fold changes in gene expression. The four treatments were non-inoculated cultivated rice (C), cultivated rice inoculated with *Magnaporthe oryzae* (C + F), non-inoculated wild rice (W), and wild rice inoculated with *M. oryzae* (W + F). (PDF 211 kb)

Additional file 7: **Figure S5.** MapMan analysis of the secondary metabolic pathways using the differentially expressed genes derived from (a) comparison C + F vs C, and (b) comparison W + F vs W. Red arrows indicate the pathways enriched in up-regulated genes. Blue and red colors indicate down- and up-regulated genes, respectively. The colored bar in each panel shows fold changes in gene expression. The four treatments were non-inoculated cultivated rice (C), cultivated rice inoculated with *Magnaporthe oryzae* (C + F), non-inoculated wild rice (W), and wild rice inoculated with *M. oryzae* (W + F). (PDF 194 kb)
