# Supplementary material for: Comparative analysis of the root transcriptomes of cultivated and wild rice varieties in response to Magnaporthe oryzae infection revealed both common and species-specific pathogen responses
Source: Rice (N Y). 2018 Apr 20;11:26. doi: 10.1186/s12284-018-0211-8 (PMC5910329; doi:10.1186/s12284-018-0211-8)
Supplement: Supplementary file 1 — Figure S1. Aerial parts of non-inoculated and inoculated cultivated and wild rice varieties. The four treatments were non-inoculated cultivated rice (C), cultivated rice inoculated with Magnaporthe oryzae (C + F), non-inoculated wild rice (W), and wild rice inoculated with M. oryzae (W + F). Black line indicates the scale bar of 1 cm. (PDF 239 kb) [file 12284_2018_211_MOESM1_ESM.pdf]

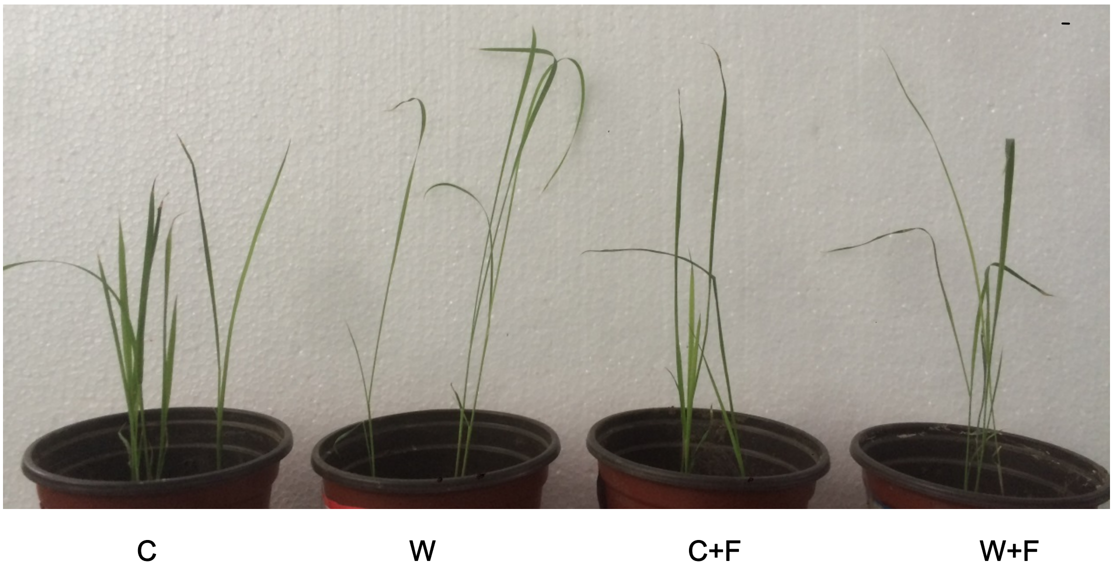

Additional file 1: Figure S1 Aerial parts of non-inoculated and inoculated cultivated and wild rice varieties. The four treatments were non-inoculated cultivated rice (C), cultivated rice inoculated with *Magnaporthe oryzae* (C+F), non-inoculated wild rice (W), and wild rice inoculated with *M. oryzae* (W+F). Black line indicates the scale bar of 1 cm.
