# Supplementary material for: Comparative analysis of the root transcriptomes of cultivated and wild rice varieties in response to Magnaporthe oryzae infection revealed both common and species-specific pathogen responses
Source: Rice (N Y). 2018 Apr 20;11:26. doi: 10.1186/s12284-018-0211-8 (PMC5910329; doi:10.1186/s12284-018-0211-8)
Supplement: Supplementary file 2 — Table S1. Summary of Illumina RNA-sequencing reads mapped to the reference genes. The four treatments were non-inoculated cultivated rice (C), cultivated rice inoculated with Magnaporthe oryzae (C + F), non-inoculated wild rice (W), and wild rice inoculated with M. oryzae (W + F). (PDF 7 kb) [file 12284_2018_211_MOESM2_ESM.pdf]

Additional file 2: Table S1 Summary of Illumina RNA-sequencing reads mapped to the reference genes. The four treatments were non-inoculated cultivated rice (C), cultivated rice inoculated with *Magnaporthe oryzae* (C+F), non-inoculated wild rice (W), and wild rice inoculated with *M. oryzae* (W+F).

**Treatment**	**Total reads in each sample(M)**	**Total base pairs in each sample(G)**	**Total clean reads in each sample(M)**	**Total mapped reads in each sample(M)**	**Percentage of reads mapped in each sample**	**Average percentage of reads mapped in each treatment**	**Average read length**
**C1**	64.645	9.6970	57.375	50.830	88.590		145.8
**C2**	64.752	9.7130	57.928	52.421	98.000	91.77	145.9
**C3**	63.555	9.5330	55.533	49.276	88.730		145.4
**C+F1**	64.078	9.6120	57.121	49.273	86.260		145.7
**C+F2**	33.887	5.0830	29.646	26.489	89.350	88.28	145.4
**C+F3**	40.453	6.0680	36.169	32.277	89.240		145.5
**W1**	38.137	5.7210	33.901	28.271	83.390		145.7
**W2**	30.544	4.5820	27.635	22.224	80.420	80.70	146
**W3**	46.214	6.9320	41.264	32.304	78.290		145
**W+F1**	52.278	7.8420	46.386	36.753	79.230		145.4
**W+F2**	62.420	9.3630	54.258	43.610	80.380	78.12	144.4
**W+F3**	63.176	9.4760	54.745	40.929	74.760		144.8
