# Supplementary material for: Comparative analysis of the root transcriptomes of cultivated and wild rice varieties in response to Magnaporthe oryzae infection revealed both common and species-specific pathogen responses
Source: Rice (N Y). 2018 Apr 20;11:26. doi: 10.1186/s12284-018-0211-8 (PMC5910329; doi:10.1186/s12284-018-0211-8)
Supplement: Supplementary file 3 — Figure S2. (a) Principal component analysis (PCA) of the total samples and (b) total mapped unigenes for the C, W, C + F and W + F groups. The four treatments were non-inoculated cultivated rice (C), cultivated rice inoculated with Magnaporthe oryzae (C + F), non-inoculated wild rice (W), and wild rice inoculated with M. oryzae (W + F). (PDF 32 kb) [file 12284_2018_211_MOESM3_ESM.pdf]

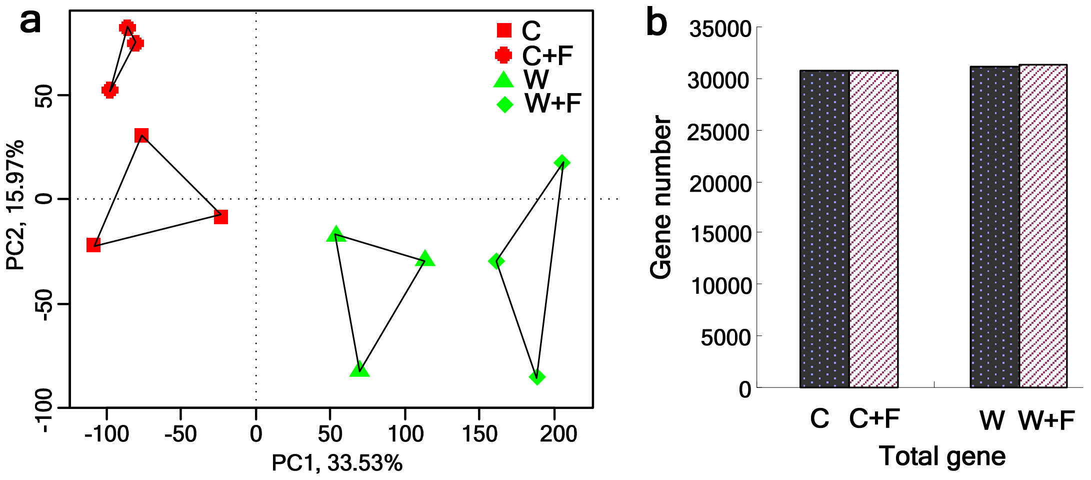

Additional file 3: Figure S2 (a) Principal component analysis (PCA) of the total samples and (b) total mapped unigenes for the C, W, C+F and W+F groups. The four treatments were non-inoculated cultivated rice (C), cultivated rice inoculated with *Magnaporthe oryzae* (C+F), non-inoculated wild rice (W), and wild rice inoculated with *M. oryzae* (W+F).
