# Supplementary material for: Comparative analysis of the root transcriptomes of cultivated and wild rice varieties in response to Magnaporthe oryzae infection revealed both common and species-specific pathogen responses
Source: Rice (N Y). 2018 Apr 20;11:26. doi: 10.1186/s12284-018-0211-8 (PMC5910329; doi:10.1186/s12284-018-0211-8)
Supplement: Supplementary file 4 — Table S2. RNA-sequencing and quantitative reverse transcription PCR (qRT-PCR) data of the verified genes. The fold-changes shown were obtained from RNA-sequencing and qRT-PCR data derived from C + F vs C comparison and W + F vs W comparison. Red, blue and black colors indicate the up- (fold-changes ≥2 with a q-value < 0.05), down-regulated (fold-changes ≤2 with a q-value < 0.05) and unchanged genes, respectively. The four treatments were non-inoculated cultivated rice (C), cultivated rice inoculated with Magnaporthe oryzae (C + F), non-inoculated wild rice (W), and wild rice inoculated with M. oryzae (W + F). (PDF 54 kb) [file 12284_2018_211_MOESM4_ESM.pdf]

Additional file 4: Table S2. RNA-sequencing and quantitative reverse transcription PCR (qRT-PCR) data of the verified genes. The fold-changes shown were obtained from RNA-sequencing and qRT-PCR data derived from C+F vs C comparison and W+F vs W comparison. Red, blue and black colors indicate the up- (fold-changes ≥2 with a q-value <0.05), down-regulated (fold-changes ≤2 with a q-value <0.05) and unchanged genes, respectively. The four treatments were non-inoculated cultivated rice (C), cultivated rice inoculated with *Magnaporthe oryzae* (C+F), non-inoculated wild rice (W), and wild rice inoculated with *M. oryzae* (W+F).

Gene ID	RNA-sequencing data				qRT-PCR data			
	C+F vs C		W+F vs W		C+F vs C		W+F vs W	
	Fold-change	q-value	Fold-change	q-value	Fold-change	q-value	Fold-change	q-value
Os06g0726200	-1.69	0.06	4.93	<0.01	-2.32	0.09	9.73	<0.01
Os12g0168700	1.46	0.06	2.29	0.04	-2.14	0.21	5.85	<0.01
Os03g0290300	-1.08	0.77	2.41	<0.01	-1.88	0.07	4.38	<0.01
Os08g0448000	1.02	0.96	2.54	<0.01	-2.64	0.24	11.79	<0.01
Os04g0229100	-1.74	<0.01	2.01	<0.01	-2.75	<0.01	4.92	<0.01
Os02g0627100	1.39	0.04	2.51	<0.01	1.52	0.08	6.50	<0.01
Os04g0483500	3.13	<0.01	-1.23	0.47	3.50	<0.01	1.41	0.08
