# Supplementary material for: Comparative analysis of the root transcriptomes of cultivated and wild rice varieties in response to Magnaporthe oryzae infection revealed both common and species-specific pathogen responses
Source: Rice (N Y). 2018 Apr 20;11:26. doi: 10.1186/s12284-018-0211-8 (PMC5910329; doi:10.1186/s12284-018-0211-8)
Supplement: Supplementary file 5 — Figure S3. (a) Total, (b) up- and (c) down-regulated differentially expressed genes (DEGs) identified in comparison W + F vs W and comparison C + F vs C by gene ontology annotation analysis. The four treatments were non-inoculated cultivated rice (C), cultivated rice inoculated with Magnaporthe oryzae (C + F), non-inoculated wild rice (W), and wild rice inoculated with M. oryzae (W + F). (PDF 15 kb) [file 12284_2018_211_MOESM5_ESM.pdf]

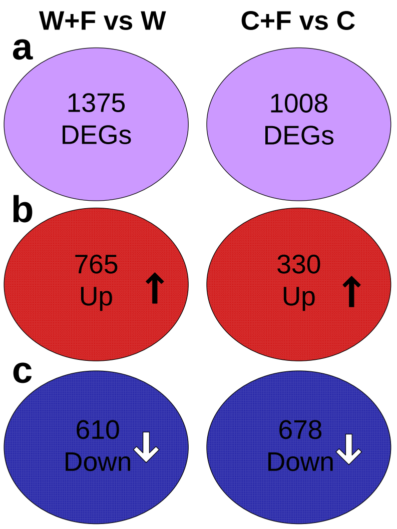

Additional file 5: Figure S3 (a) Total, (b) up- and (c) down-regulated differentially expressed genes (DEGs) identified in comparison W+F vs W and comparison C+F vs C by gene ontology annotation analysis. The four treatments were non-inoculated cultivated rice (C), cultivated rice inoculated with *Magnaporthe oryzae* (C+F), non-inoculated wild rice (W), and wild rice inoculated with *M. oryzae* (W+F).
