# Supplementary material for: Comparative analysis of the root transcriptomes of cultivated and wild rice varieties in response to Magnaporthe oryzae infection revealed both common and species-specific pathogen responses
Source: Rice (N Y). 2018 Apr 20;11:26. doi: 10.1186/s12284-018-0211-8 (PMC5910329; doi:10.1186/s12284-018-0211-8)
Supplement: Supplementary file 7 — Figure S5. MapMan analysis of the secondary metabolic pathways using the differentially expressed genes derived from (a) comparison C + F vs C, and (b) comparison W + F vs W. Red arrows indicate the pathways enriched in up-regulated genes. Blue and red colors indicate down- and up-regulated genes, respectively. The colored bar in each panel shows fold changes in gene expression. The four treatments were non-inoculated cultivated rice (C), cultivated rice inoculated with Magnaporthe oryzae (C + F), non-inoculated wild rice (W), and wild rice inoculated with M. oryzae (W + F). (PDF 194 kb) [file 12284_2018_211_MOESM7_ESM.pdf]

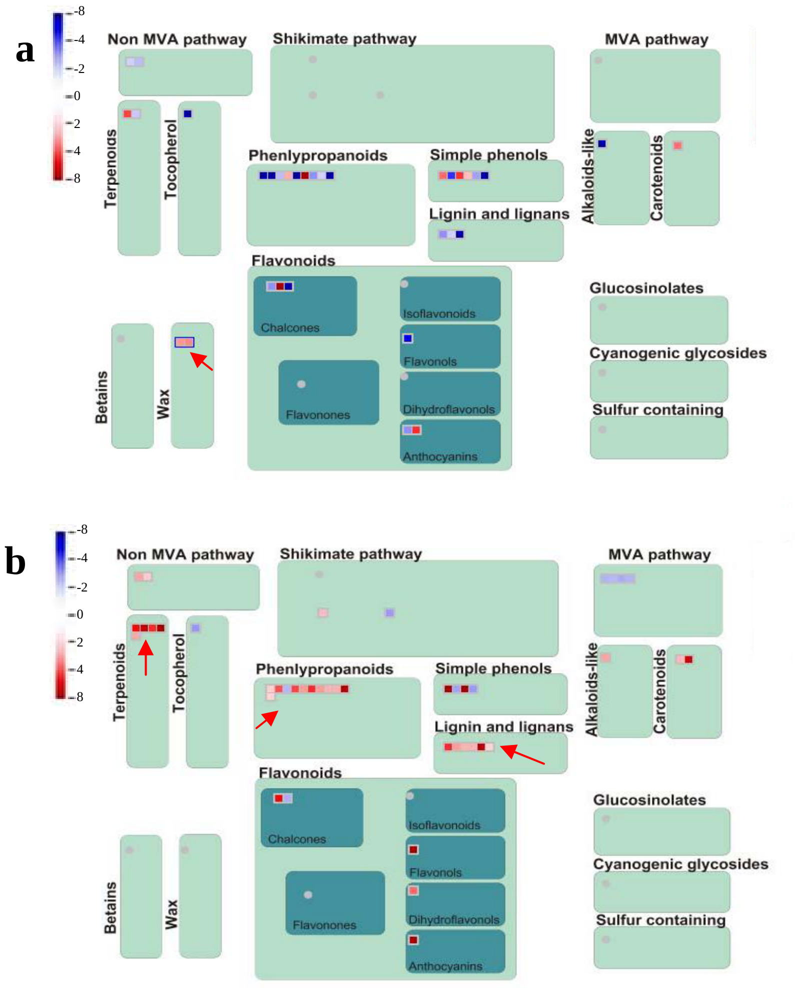

Additional file 7: Figure S5 MapMan analysis of the secondary metabolic pathways using the differentially expressed genes derived from (a) comparison C+F vs C, and (b) comparison W+F vs W. Red arrows indicate the pathways enriched in up-regulated genes. Blue and red colors indicate down- and up-regulated genes, respectively. The colored bar in each panel shows fold changes in gene expression. The four treatments were non-inoculated cultivated rice (C), cultivated rice inoculated with *Magnaporthe oryzae* (C+F), non-inoculated wild rice (W), and wild rice inoculated with *M. oryzae* (W+F).
